# Malnutrition Is Associated With Impaired Functional Status in Older People Receiving Home Care Nursing Service

**DOI:** 10.3389/fnut.2021.684438

**Published:** 2021-06-14

**Authors:** Luana Lemos Leão, Knut Engedal, Renato Sobral Monteiro-Junior, Gro Gujord Tangen, Maria Krogseth

**Affiliations:** ^1^Graduate Program of Health Sciences, State University of Montes Claros, Montes Claros, Brazil; ^2^Department of Geriatric Medicine, Oslo University Hospital, Oslo, Norway; ^3^Norwegian National Advisory Unit on Ageing and Health, Vestfold Hospital Trust, Tønsberg, Norway; ^4^Graduate Program of Medicine (Neurology/Neuroscience), Federal Fluminense University, Rio de Janeiro, Brazil; ^5^Old Age Psychiatry Research Network, Telemark Hospital Trust and Vestfold Hospital Trust, Tønsberg, Norway; ^6^Department of Nursing and Health Sciences, University of South-Eastern Norway, Drammen, Norway

**Keywords:** nutritional status, older adult, nutritional assessment, frailty, comorbididites

## Abstract

**Objective:** This study aimed to explore the magnitude and significance of associations among nutritional status, functional status, comorbidities, age, and gender in older adults receiving assistance from the in-home nursing care service.

**Method:** In this cross-sectional study, 210 home-dwelling persons 65 years or older who received in-home nursing care service were evaluated. Demographic variables, nutritional status, comorbidities, and the dependency levels of activities of daily living were analyzed. To assess the correlation among the factors that influence nutritional status, a theoretical model was developed and adjusted using the path analysis model.

**Results:** The primary finding is that functional status is directly associated with nutritional status (β = 0.32; *p* < 0.001) and severity of comorbidities is indirectly associated with nutritional status (β = −0.07; *p* < 0.017).

**Conclusion:** The elicited outcomes in this study reinforce the concept that nutritional status is linked with functional status in older adults receiving in-home care nursing service.

## Introduction

According to demographic data, the older adult population has been increasing dramatically in the last 50 years, and it will increase further in the year to come. In 2019, 703 million people were aged 65 years or above in the world. In 2,050, the number is expected to increase to 1.5 billion, meaning that one in every six people worldwide will be aged 65 years or over (1).

Aging is defined as an individual, sequential and cumulative series of physiological changes that occur in an organism over time, resulting in progressive deterioration of functioning, increased vulnerability to disease, and reduced viability (2). Aging is in most people accompanied by multimorbidity and physically debilitating conditions such as sarcopenia, frailty, dementia, and/or malnutrition (3).

Older people may suffer from frailty and malnutrition at the same time. Previous studies have shown a strong association between physical frailty and risk of malnutrition and malnutrition in older adults (4, 5). Thus, a critical agent for healthy aging is an ideal nutritional status. There are several causes for this: reduced appetite and energy expenditure, fluid and electrolyte imbalance, altered levels of cytokines and hormones, delayed gastric emptying, and an impaired sense of smell and taste. Moreover, pathologic changes of aging such as chronic disease, depression, cognitive impairment, multiple morbidities, and polypharmacy play an important role in the complex etiology of malnutrition in older adults (6–8). Hence, studies worldwide have shown the importance of an adequate nutritional status in this critical period of life (9, 10).

Early identification of older adults who are at risk for insufficient caloric intake and nutrient adequacy, termed nutritional risk, or malnutrition, is paramount to maintaining health, independence, quality of life, and longevity (11). The prevalence of malnutrition is reported to be 10–50% in different populations of older people in need of health care services (8, 12). A previous study of older patients receiving domiciliary health care in Norway reported that 41% had dementia and 72% had neuropsychiatric symptoms such as depression, apathy, and anxiety (13). These findings underline that this population is very vulnerable and may consist of older frail people at risk for malnutrition, or even present insufficient caloric intake. Therefore, this study aimed to explore the magnitude and significance of associations among nutritional status, functional status, comorbidities, age, and gender in older adults receiving assistance from the in-home nursing care service in Norway.

## Methods

### Study Design and Population

This study is part of the Capturing Acute and Social Care in Dependent Elders (CASCADE), a prospective cross-sectional study on home-dwelling persons 65 years and older who received in-home nursing care service at least once a week in 2016 in a small city (Sandefjord) in the southeast of Norway. Sample size calculation was carried out using information regarding the Norwegian older population receiving home care nursing services (*N* = 140,000 individuals) (14), the confidence level (95%), confidence interval (5%), and the proportion of potential events (prevalence of malnutrition = average 19%) (15). Data were inserted in the Sample Size Calculator, a tool designed by the Australian Bureau of Statistics (16). The estimated sample was established (*n* = 237 participants). To be included, the patients must have had in-home nursing care service for 4 weeks or more to ensure the service's knowledge about the patients' function in a stable situation. From the 588 persons aged >65 years receiving home care nursing in this community, the head nurses continuously selected patients by alphabetical order that fulfilled the inclusion criteria. Patients were not included if they suffered from a terminal illness (life expectancy <2 weeks); if they had a diagnosis of Lewy-body dementia (fluctuations of symptoms make the diagnostic of delirium challenging in these patients); if suffering from chronic disease that has led to need of assistance from the in-home nursing care service before the age of 65 years; or if need of in-home nursing care service due to substance abuse or psychiatric disease (not dementia) that arise before the age of 65 years adults. Although the sampling calculation established *n* = 237 participants, due to the exclusion criteria aforementioned, in all, 210 older persons were included, of whom 138 (65.7%) were women. The mean age of the entire sample was 84.5 years (± 8.3), in women it was 85.9 years (± 7.85) and in men, it was 81.4 years (± 8.25). The Regional Committee for Ethics in Medical and Health Research and the Data Protection Officer approved the project (2014/1972).

#### Assessment

The visits in the participants' own homes for collecting data were performed by trained health professionals. The following information was collected: demographic variables and for nutritional status, the full version of the Mini Nutritional Assessment (MNA) was performed. The MNA comprises 18 items grouped in four sections: anthropometric assessment (weight, height, and weight loss); general assessment (lifestyle, medication use, and mobility); dietary assessment (number of meals, food and fluid intake, and autonomy of feeding); and subjective assessment (self-perception of health and nutrition status). Each response has a numerical value and contributes to the final score, which has a maximum value of 30. A score of 24 or higher indicates satisfactory nutritional status; a score of 17 to 23.5 indicates a risk of malnutrition; a score below 17 indicates protein-energy malnutrition (17, 18). Charlson Comorbidity Index (CCI) was calculated according to the comorbidities reported, and the severity scale of CCI was used to classify the severity of comorbidity. The CCI is primarily based on medical record review so as to assign weights for a number of major comorbid conditions. For each condition, the weight is approximately equal to the one-year relative risk of death for that condition. The index score is the total of assigned weights and represents a measure of the burden of comorbid disease (19). The Barthel Index (BI) was applied to measure the functional status. The BI measures the severity of impairment of tasks, such as toilet use, eating, dressing, and climbing stairs. The maximum score is 20 with higher scores indicating better performance and a higher degree of independence in activities of daily living (20).

#### Statistical Analyses

**Figure 1:**
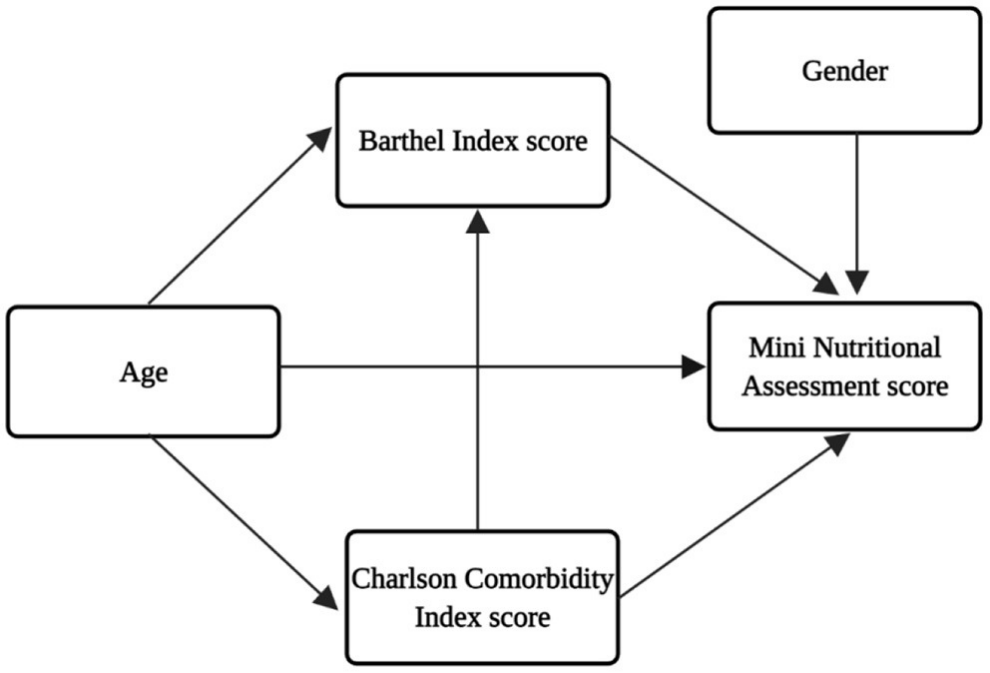
Theoretical model for the interrelationships of the factors that influence nutritional status (MNA score).

Continuous variables were summarized as mean and standard deviation (SD). Chi-square, One-way and Two-way ANOVA were used to compare variables of subgroups. We grouped the participants into two age-groups, <85 and ≥85 years old. The 85-year cutoff point was adopted because people aged 85 years and above are considered to be the “oldest old,” and this group presents increased risks of malnutrition, multimorbidity, and disability (21). Categorical variables were described by their frequency distribution and chi-square tests were executed for comparison of categorical variables. The following numerical variables were tested for normality: MNA (sk = 0.82; ku = 0.36); Age (sk = −0.38; ku = −0.71), Years of Education (sk = 1.15; ku = 1.57), BI (sk = −1.37; ku = 1.65 and CCI (sk = 0.99; ku = 1.02). Values of skew (sk) > 3 and/or kurtosis (ku)> 7 were considered indicators of a violation of the assumption of normality (22). According to evidence, the nutritional status in older adults is related to a great range of factors, including gender, age, comorbidities, and functional status (23–26). To assess the correlations among the factors that influence nutritional status in the investigated sample, a theoretical model illustrated in Figure 1 was developed.

The model was adjusted using the path analysis. Direct and indirect effects were estimated using standardized coefficients, adopting a significance level of 0.05. Standardized coefficients with values 0.10–0.29, 0.30–0.49, and >0.50 were interpreted as small, medium, and large effects, respectively (22).

The Bentler's comparative fit index (CFI), the goodness of fit index (GFI), and the Tucker-Lewis index (TLI) were used to assess the quality of the adjustments of the measurement and structural models. These indexes indicate a good adjustment when values >0.90 are reached. The root mean squared error of approximation (RMSEA) was also used, whose value below 0.10 was considered an indicator of reasonable adjustment. In addition, the absolute index χ^2^/df was adopted, since this indicates an acceptable adjustment for a value <3 (22, 27–29). The IBM SPSS 23.0 software was used to perform the descriptive analyzes. The software IBM SPSS AMOS 23.0 and R 3.5.0 were used to adjust the model.

## Results

The proportion of females was higher than males. Concerning the comorbidities, 29.5 and 22.4% had dementia and heart failure, respectively. A lower CCI score mean was found among women (2.38 ± 1.86), while men presented a higher score (3.09 ± 2.18; *p* = 0.014). According to the MNA score in this study, 15.3% of men and 16.8% of females were malnourished, and most (59.7% of men and 59.1% of women) of the participants were at risk of malnutrition. Table 1 shows the descriptive statistics and characteristics of the participants, whereas Tables 2, 3 show the patients' functional status, the severity of comorbidities, and prevalence of malnutrition and at risk for malnutrition separated by age groups (<85 years vs. ≥85 years). The highest CCI score (3.05 ± 2.18; *p* = 0.002) was seen in the group <85 years. However, there was no difference in the CCI score according to the nutritional status in both age groups (*p* = 0.154). Among the oldest old men, 40% were malnourished, whereas 60% of women of the same age group were malnourished. Among the oldest-old group, 21.7% of the men, and 78.3% of the women were at risk of malnutrition. No significant difference in nutritional status was found between men and women (χ^2^ = 0.080; *p* = 0.961) or among the marital status (χ^2^ = 1,926; *p* = 0.926).

**Table 1:** Sex, years of education, marital status and comorbidities of the participants.

**Categorical variables**	**N**	**%**
**Sex**		
Male	72	34.3
Female	138	65.7
**Years of education**		
≤10	142	67.6
>10	68	32.4
**Age**		
<85	101	48.1
≥85	109	51.9
**Marital status**		
Married/cohabitant	66	31.4
Divorced	31	14.7
Widowed	107	51.0
Single	06	2.9
**Comorbidity prevalence**		
Myocardial Infarction	38	18.1
Heart failure	47	22.4
Peripheral vascular disease	19	9
Transient ischemic attack	42	20
Dementia	62	29.5
Chronic obstructive pulmonary disease	42	20
Connective tissue disease	33	15.7
Peptic ulcer disease	28	13.3
Mild liver disease	2	1
Diabetes	41	19.5
Diabetes with end-organ damage	9	4.3
Hemiplegia	23	11
Moderate-to-severe renal disease	23	11
Tumor	23	11
Metastatic solid tumor	7	3.3
Leukemia	1	0.5

*Values are expressed as n and % for categorical variables*.

**Table 2:** Distribution of participants' characteristics by age groups.

**Variables**	**Age**				
	**<85**		**≥85**		
	***n*** **= 101**		***n*** **= 108**		
	**Mean**	**SD**	**Mean**	**SD**	**p**
Age (years)	77.29	5.37	91.13	3.57	**<0.001**
Years of education	10	3.43	9.514	3.27	0.325
Barthel Index (score)	15.78	4.03	15.84	3.34	0.906
**Charlson Comorbidity Index (score)**	3.05	2.18	2.21	1.75	**0.002**
Malnourished	3.84	2.47	2.13	1.59	0.154^*^
At risk of malnutrition	3.09	2.31	2.30	1.87	
Normal nutritional status	2.44	1.45	2.00	1.57	

*Values are expressed as mean and SD for continuous variables*.

* *P-value of the comparison between the nutritional status groups. P-value results are from ANOVA. Bold values indicate statistical significance*.

**Table 3:** Participants' nutritional status by age according to sex and marital status.

**Nutritional status**	**Variables**	***n***	**%**	***n***	**%**	**Significance**
		**<85**		**≥85**		
		***n*** **= 101**		***n*** **= 108**		
Malnourished	Men	5	26.3	6	40	0.316 χ^2^ = 0,717
	Women	14	73.7	9	60.0	
	Married/cohabitant	6	31.6	5		0.086 χ^2^ = 4,911
	Divorced	5	26.3	0	0.0	
	Widowed	8	42.1	10	66.7	
At risk of malnutrition	Men	28	50.9	15	21	**0.001** χ^2^ = 11,496
	Women	27	49.1	54	78.3	
	Married/cohabitant	24	43.6	16		**0.013** χ^2^ = 10,825
	Divorced	10	18.2	8	11.6	
	Widowed	18	32.7	43	62.3	
	Single	3	5.5	2	2.9	
Normal	Man	12	44.4	6	25	0.123 χ^2^ = 2,104
	Women	15	55.6	18	75.0	
	Married/cohabitant	8	29.6	7		0.096 χ^2^ = 6,338
	Divorced	7	25.9	1	4.2	
	Widowed	11	40.7	16	66.7	
	Single	1	3.7	0	0.0	

*Values are expressed as n and % for categorical variables. Significance according to the Chi-square test. Bold font indicates statistical significance*.

**Figure 2:**
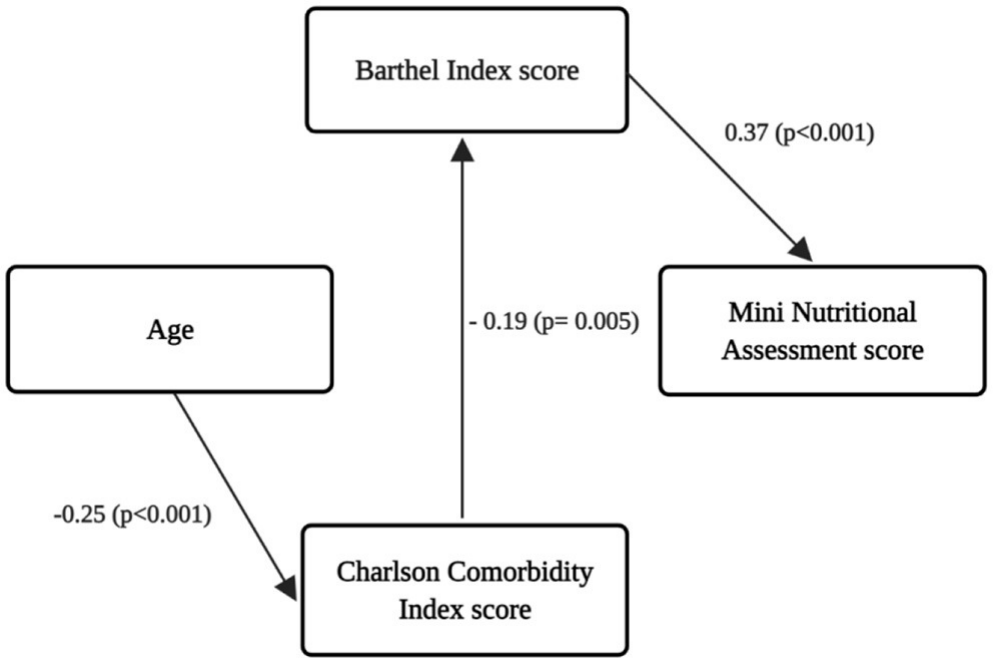
Adjusted model showing the significant associations among variables.

Figure 2 displays the results of the path analysis, whose adjustment indexes were considered satisfactory: χ^2^/df = 2.416; CFI = 0.974; GFI = 0.861; TLI = 0.800; RMSEA = 0.08 (CI90% 0.03–0.13; *p* = 0.134). The variables considered in the adjusted model were Age, CCI and BI. The BI score was the only variable with a medium direct positive and significant effect on the MNA score (β = 0.37; *p* < 0.001). The CCI score had a small negative direct effect on BI score (β = −0.19; *p* < 0.005) and age had a negative small effect on the CCI score (β = −0.25; *p* < 0.001). The CCI score also had an indirect effect on the MNA score (β = −0.07; *p* < 0.017). This indirect effect was calculated by multiplying the β result of the BI and the MNA β. The other trajectories showed in the theoretical model (Figure 1) were not statistically significant and were removed from the adjusted model.

## Discussion

The current study examined the associations among nutritional status, functional status, comorbidities, age, and gender in older adults receiving assistance from the in-home nursing care service. The primary finding is that functional status is directly associated with nutritional status and comorbidities are indirectly associated with nutritional status.

Malnutrition is an important public problem observed more frequently in older people compared to the general population (30). This study showed that 15.3% of men and 16.8% of females were malnourished, and most of the participants were at risk of malnutrition. Similarly, Yamamoto et al. (31) also evaluated the nutritional status of older patients (mean age of 84 years old) receiving home care in Japan. According to the MNA evaluation, 18.6 and 49.5% of the participants in the Japanese study were malnourished and at risk of malnutrition, respectively. It has been reported that health, environmental, and social factors or determinants are connected with the risk of malnutrition in older people (32). Especially the widowed older adults are the most malnourished or at the most risk of malnutrition (33). Our study did not find a significant association between marital and nutritional status. However, 62.3% of the widowed oldest-old participants were at risk of malnutrition. According to Besora-Moreno et al. (33), widowhood is related to poor eating habits and less enjoyment of eating.

Although previous research showed a significant correlation between the nutritional status and female gender (34), we did not find any significant relationship between nutritional status and the two age groups of men and women. A similar result was found by Asamane et al. (35) in the United Kingdom. This could be because in general, the women presented fewer comorbidities when compared to men in our study. Moreover, Norway is currently one of the most egalitarian countries in the world according to the Global Gender Gap Index Ranking (36). Thus, it could be assumed that females had fairly equal access to food and therefore eat diets similar to their male counterparts.

Our study also showed an indirect effect of the CCI on the MNA score. The correlation between malnutrition and comorbidities is well-known, and previous studies reported that malnourished older people have higher CCI scores (37, 38). The most prevalent comorbidity found in the participants of the current study was dementia (29.5%). Sanders et al. (39) examined the association of nutritional status and rate of cognitive and functional decline in older adults. The findings showed that malnutrition is associated with more severe symptoms of dementia, and those older people with higher MNA scores would likely experience higher overall cognitive abilities over the course of dementia than those with lower MNA scores.

Furthermore, in this study, age had a direct negative effect on the CCI score. This result diverges from Magdalini et al. (40) study, which shows that there is a significant correlation between older age and increased CCI. However, as our population was receiving in-home nursing services, which is already identified as a very frail group (41), the younger older people presented more severe comorbidities when compared to the oldest old people. Moreover, the other hypothesis is that the oldest old group could be the fittest due to genetic factors and a lifelong favorable lifestyle. However, we did not explore those other factors.

The CCI score had a direct negative effect on functional status, as expected. Mayoral et al. (42) evaluated older adults with osteoporotic hip fracture in order to verify the influence of comorbidities and cognitive impairment on the physical recovery of those patients, during the first year following the fracture. The results demonstrated that CCI clearly influenced the functional status recovery. Low values of CCI indexes resulted in better BI recovery (42).

Malnutrition has been linked with poor functional status as it is an important contributor toward increased vulnerability for developing negative health outcomes, loss of independence, and mortality (18, 43). Meal preparation and eating disabilities seem to be the main cause of malnutrition in older adults with poor functional status (44). Our study showed a positive and significant effect of functional status on the MNA score. Indeed, these findings are in agreement with previous studies. MNA scores and functional status are positively correlated, and it is shown that both nutritional and functional status worsens with age (38, 45, 46). One possible explanation of how nutritional status affects the functional status could be that low energy and especially low protein intake leads to a loss of muscles and strength and consequent loss of daily function (47). Prevention and treatment of age-related disorders can be done through nutritional interventions, which consider both material and human resources required, such as attendance of qualified nutritionists to routinely conduct effective evaluations and interventions required for maintenance of proper health of older adults receiving home care nursing service.

A strength of this study is the availability of information regarding nutrition and health status which was obtained by standardized questionnaires and geriatric assessment tools. This research, however, has some limitations. First, the cross-sectional design, which limits conclusions regarding causal effects or intraindividual changes. Second, the inclusion of patients in alphabetical order may represent a bias for the participants' selection. Third, the comorbidity assessment was based on information from medical records only. Furthermore, the muscle mass index, physical performance, and polypharmacy were not available to all participants, which make insertion as control variables difficult. Finally, dietary intake was not assessed; therefore, the reasons for malnutrition risk could not be identified.

## Conclusion

In this study, 15.3% of men and 16.8% of women were malnourished and almost 60% of both sexes were at risk of malnutrition. The elicited outcomes reinforce that severity of comorbidities had a direct impact on functional status, and functional status, in turn, had a direct effect on the nutritional status of older adults receiving in-home nursing care service.

## Data Availability Statement

The raw data supporting the findings of this study are available from the corresponding author, Maria Krogseth, upon request.

## Ethics Statement

The studies involving human participants were reviewed and approved by the Regional Committee for Ethics in Medical and Health Research and the Data Protection Officer approved the project (2014/1972). The patients/participants provided their written informed consent to participate in this study.

## Author Contributions

LL and MK: conceptualization/study design, methodology, manuscript preparation, review, editing, supervision, project administration, and funding acquisition. LL and RM-J: statistical analysis, review, and editing. KE and GT: data curation and review. All authors contributed to the article and approved the submitted version.

## Conflict of Interest

The authors declare that the research was conducted in the absence of any commercial or financial relationships that could be construed as a potential conflict of interest.

## References

[1] United Nations, Department of Economic and Social Affairs, Population Division. World Population Ageing 2019 (ST/ESA/SER.A/444). Available online at: https://www.un.org/development/desa/pd/sites/www.un.org.development.desa.pd/files/files/documents/2020/Jan/un_2019_worldpopulationageing_report.pdf (accessed January 15, 2021).

[2] Phillip JM Aifuwa I Walston J Wirtz D. The mechanobiology of aging. Annu Rev Biomed Eng. (2015) 17:113–41. 10.1146/annurev-bioeng-071114-040829 26643020 PMC4886230

[3] Gómez-Gómez ME Zapico SC. Frailty, cognitive decline, neurodegenerative diseases and nutrition interventions. Int J Mol Sci. (2019) 11:2842. 10.3390/ijms20112842 31212645 PMC6600148

[4] BollweinJVolkertDDiekmannRKaiserMJUterWVidalK. Nutritional status according to the mini nutritional assessment (MNA) and frailty in community dwelling older persons. J Nutr Health Aging. (2013) 17:351–6. 10.1007/s12603-013-0034-723538658

[5] Eyigor S Kutsal YG Duran EM Huner B Paker N Durmus B. Frailty prevalence and related factors in the older adult-FrailTURK Project. Age. (2015) 3:9791. 10.1007/s11357-015-9791-z 25948502 PMC4422824

[6] Ahmed T Haboubi N. Assessment and management of nutrition in older people and its importance to health. Clin Interv Aging. (2010) 5:207–16. 10.2147/CIA.S9664 20711440 PMC2920201

[7] Porter Starr KN McDonald SR Bales CW. Nutritional vulnerability in older adults: a continuum of concerns. Curr Nutr Rep. (2015) 2:176–84. 10.1007/s13668-015-0118-6 26042189 PMC4445877

[8] Engelheart S Brummer R. Assessment of nutritional status in the elderly: a proposed function-driven model. Food Nutr Res. (2018) 62. 10.29219/fnr.v62.1366 29720931 PMC5917421

[9] Choe YR Joh JY Sunwoo D Kim YP. Interaction between frailty and nutritional status on mortality and long-term hospitalization in older Koreans: a retrospective analysis of data from the 2008 survey on health and welfare status of the elderly in Korea. Arch Gerontol Geriatr. (2018) 76:106–13. 10.1016/j.archger.2018.01.011 29486379

[10] Karakas N Bentli R Firinci B Zabci B. Investigation of the relationship between depression and nutritional status of elderly patients in home care. J Surg Med. (2019) 3:829–32. 10.28982/josam.650233

[11] Kim DE Lim H-S Ahn H Kim YS Park YK. Sex differences in the association between living environmental factors and nutritional status in community-dwelling elderly koreans. Int J Environ Res Public Health. (2020) 17:6034. 10.3390/ijerph17176034 32825086 PMC7504555

[12] Kaiser MJ Bauer JM Rämsch C Uter W Guigoz Y Cederholm T. Frequency of malnutrition in older adults: a multinational perspective using the mini nutritional assessment. J Am Geriatr Soc. (2010) 58:1734–8. 10.1111/j.1532-5415.2010.03016.x 20863332

[13] Wergeland JN Selbæk G Høgset LD Söderhamn U Kirkevold Ø. Dementia, neuropsychiatric symptoms, and the use of psychotropic drugs among older people who receive domiciliary care: a cross-sectional study. Int Psychogeriatr. (2014) 26:383–91. 10.1017/S1041610213002032 24252377

[14] Statistisk, Sentralbyrå. Available online at: https://www.ssb.no/en/statbank/table/11642/ (accessed April 21, 2021).

[15] Van Nie-Visser N Meijers J Schols J Lohrmann C Bartholomeyczik S Spreeuwenberg M. Which characteristics of nursing home residents influence differences in malnutrition prevalence? An international comparison of The Netherlands, Germany and Austria. Br J Nutr. (2014) 111:1129–36. 10.1017/S0007114513003541 24246053

[16] Australian Bureau of Statistics. Sample Size Calculator. Available online at: https://www.abs.gov.au/websitedbs/d3310114.nsf/home/sample+size+calculator (accessed January 15, 2021).

[17] Vellas B Guigoz Y Garry PJ Nourhashemi F Bennahum D Lauque S. The mini nutritional assessment (MNA) and its use in grading the nutritional state of elderly patients. Nutrition. (1999) 15:116–22. 10.1016/S0899-9007(98)00171-3 9990575

[18] Guigoz Y. The mini nutritional assessment (MNA) review of the literature–what does it tell us? J Nutr Health Aging. (2006) 10:466–85. 17183419

[19] Charlson ME Pompei P Ales KL MacKenzie CR. A new method of classifying prognostic comorbidity in longitudinal studies: development and validation. J Chronic Dis. (1987) 5:373–83. 10.1016/0021-9681(87)90171-8 3558716

[20] Mahoney FI Barthel DW. Functional evaluation: the barthel index. Md State. Med J. (1965) 14:61–5. 10.1037/t02366-000 14258950

[21] Granic A Mendonça N Hill TR Jagger C Stevenson EJ Mathers JC. Nutrition in the very old. Nutrients. (2018) 3:26. 10.3390/nu10030269 PMC5872687 29495468

[22] Kline RB. Principles and Practice of Structural Equation Modeling. 2th ed. St. New York, NY: The Guilford Press (2005).

[23] Nambooze J Fujimura M Inaoka T. Nutritional status and functional capacity of community-dwelling elderly in Southern Laos. Environ Health Prev Med. (2014) 2:143–50. 10.1007/s12199-013-0367-1 24218019 PMC3944031

[24] Agarwalla R Saikia AM Baruah R. Assessment of the nutritional status of the elderly and its correlates. J Family Community Med. (2015) 1:39–43. 10.4103/2230-8229.149588 25657610 PMC4317993

[25] Sugita Y Miyazaki T Shimada K Shimizu M Kunimoto M Ouchi S. Correlation of nutritional indices on admission to the coronary intensive care unit with the development of delirium. Nutrients. (2018) 11:1712. 10.3390/nu10111712 30413062 PMC6267104

[26] Buntinx F Niclaes L Suetens C Jans B Mertens R Van den Akker M. Evaluation of Charlson's comorbidity index in elderly living in nursing homes. J Clin Epidemiol. (2002) 11:1144–7. 10.1016/S0895-4356(02)00485-7 12507679

[27] Byrne B. Structural Equation Modelling With AMOS: Basic Concepts, Applications and Programming. Mahwah: Lawrence Erlbaum (2001).

[28] Hair JF Anderson RE Tatham RL Black WC. Análise Multivariada De Dados. Tradução: Adonai Schlup Sant'Anna e Anselmo Chaves Neto. 5th ed. St. Porto Alegre: Bookman (2005).

[29] Marôco J. Análise De Equações Estruturais: Fundamentos Teóricos, Software & Aplicações. 2nd ed. St. Lisboa: ReportNumber (2014).

[30] Suominen M Muurinen S Routasalo P Soini H Suur-Uski I Peiponen A. Malnutrition and associated factors among aged residents in all nursing homes in Helsinki. Eur J Clin Nutr. (2005) 4:578–83. 10.1038/sj.ejcn.1602111 15744328

[31] Yamamoto K Tsuji T Yamasaki K Momoki C Yasui Y Habu D. Scoring methods used in the dietary variety score survey to predict malnutrition among older patients receiving home care. Int J Older People Nurs. (2020) 3:e12301. 10.1111/opn.12301 32196974

[32] Corish CA Bardon LA. Malnutrition in older adults: screening and determinants. Proc Nutr Soc. (2019) 3:372–9. 10.1017/S0029665118002628 30501651

[33] Besora-Moreno M Llauradó E Tarro L Solà R. Social and economic factors and malnutrition or the risk of malnutrition in the elderly: a systematic review and meta-analysis of observational studies. Nutrients. (2020) 3:737. 10.3390/nu12030737 32168827 PMC7146387

[34] Mantzorou M Vadikolias K Pavlidou E Serdari A Vasios G Tryfonos C. Nutritional status is associated with the degree of cognitive impairment and depressive symptoms in a Greek elderly population. Nutr Neurosci. (2020) 3:201–9. 10.1080/1028415X.2018.1486940 29914306

[35] Asamane EA Greig CA Thompson JL. The association between nutrient intake, nutritional status and physical function of community-dwelling ethnically diverse older adults. BMC Nutr. (2020) 6:36. 10.1186/s40795-020-00363-6 32864152 PMC7447572

[36] Weforum. World Economic Forum. Global Gender Gap Report. (2020). Available online at: https://www.weforum.org/reports/gender-gap2020-report-100-years-pay-equality (accessed January 15, 2021).

[37] Tsuji T Yamamoto K Yamasaki K. Lower dietary variety is a relevant factor for malnutrition in older Japanese home-care recipients: a cross-sectional study. BMC Geriatr. (2019) 1:197. 10.1186/s12877-019-1206-z 31349800 PMC6659217

[38] Miettinen M Tiihonen M Hartikainen S Nykänen I. Prevalence and risk factors of frailty among home care clients. BMC Geriatr. (2017) 1:266. 10.1186/s12877-017-0660-8 29149866 PMC5693585

[39] Sanders C Behrens S Schwartz S Wengreen H Corcoran CD Lyketsos CG. Nutritional status is associated with faster cognitive decline and worse functional impairment in the progression of dementia: the cache county dementia progression study. J Alzheimers Dis. (2016) 52:33–42. 10.3233/JAD-150528 26967207 PMC5318140

[40] Magdalini V Petros I Constantinos T Emmanouil P Christos K George K. Age, comorbidities and fear of fall: mortality predictors associated with fall-related fractures. Maedica. (2020) 1:18–23. 10.26574/maedica.2020.15.1.18 32419856 PMC7221284

[41] Næss G Kirkevold M Hammer W Straand J Wyller TB. Nursing care needs and services utilised by home-dwelling elderly with complex health problems: observational study. BMC Health Serv Res. (2017) 1:645. 10.1186/s12913-017-2600-x 28899369 PMC5596938

[42] Mayoral AP Ibarz E Gracia L Mateo J Herrera A. The use of Barthel index for the assessment of the functional recovery after osteoporotic hip fracture: one year follow-up. PLoS ONE. (2019) 2:e0212000. 10.1371/journal.pone.0212000 30730973 PMC6366714

[43] Boulos C Salameh P Barberger-Gateau P. Malnutrition and frailty in community dwelling older adults living in a rural setting. Clin Nutr. (2016) 1:138–43. 10.1016/j.clnu.2015.01.008 25649256

[44] Bakhtiari A Pourali M Omidvar S. Nutrition assessment and geriatric associated conditions among community dwelling Iranian elderly people. BMC Geriatr. (2020) 1:278. 10.1186/s12877-020-01668-8 32762725 PMC7409695

[45] SchraderEBaumgärtelCGueldenzophHStehlePUterWSieberCC. Nutritional status according to mini nutritional assessment is related to functional status in geriatric patients–independent of health status. J Nutr Health Aging. (2014) 3:257–63. 10.1007/s12603-013-0394-z24626752

[46] Villafañe JH Pirali C Dughi S Testa A Manno S Bishop MD. Association between malnutrition and barthel index in a cohort of hospitalized older adults article information. J Phys Ther Sci. (2016) 2:607–12. 10.1589/jpts.28.607 27064250 PMC4793019

[47] Tanimoto Y Watanabe M Sun W Tanimoto K Shishikura K Sugiura Y. Association of sarcopenia with functional decline in community-dwelling elderly subjects in Japan. Geriatr Gerontol Int. (2013) 4:958–63. 10.1111/ggi.12037 23452074

